# Development and validation testing of a short nutrition questionnaire to identify dietary risk factors in preschoolers aged 12–36 months

**DOI:** 10.3402/fnr.v59.27912

**Published:** 2015-06-08

**Authors:** Niamh Rice, Helena Gibbons, Breige A. McNulty, Janette Walton, Albert Flynn, Michael J. Gibney, Anne P. Nugent

**Affiliations:** 1 Previs Healthcare Ltd, Kilmacanogue, Wicklow, Ireland; 2 UCD Institute of Food and Health, University College Dublin, Belfield, Dublin 4, Ireland; 3 School of Food and Nutritional Sciences, University College Cork, Cork, Ireland

**Keywords:** preschool children, toddlers, nutrient-poor diets, dietary quality, screening tools, nutritional risk

## Abstract

**Background:**

Although imbalances in dietary intakes can have short and longer term influences on the health of preschool children, few tools exist to quickly and easily identify nutritional risk in otherwise healthy young children.

**Objectives:**

To develop and test the validity of a parent-administered questionnaire (NutricheQ) as a means of evaluating dietary risk in young children (12–36 months).

**Design:**

Following a comprehensive development process and internal reliability assessment, the NutricheQ questionnaire was validated in a cohort of 371 Irish preschool children as part of the National Preschool Nutrition Survey. Dietary risk was rated on a scale ranging from 0 to 22 from 11 questions, with a higher score indicating higher risk.

**Results:**

Children with higher NutricheQ scores had significantly (*p*<0.05) lower mean daily intakes of key nutrients such as iron, zinc, vitamin D, riboflavin, niacin, folate, phosphorous, potassium, carotene, retinol, and dietary fibre. They also had lower (*p*<0.05) intakes of vegetables, fish and fish dishes, meat and infant/toddler milks and higher intakes of processed foods and non-milk beverages, confectionery, sugars and savoury snack foods indicative of poorer dietary quality. Areas under the curve values of 84.7 and 75.6% were achieved for ‘medium’ and ‘high’ dietary risk when compared with expert risk ratings indicating good consistency between the two methods.

**Conclusion:**

NutricheQ is a valid method of quickly assessing dietary quality in preschoolers and in identifying those at increased nutritional risk.

**In Context:**

Analysis of data from national food and nutrition surveys typically identifies shortfalls in dietary intakes or quality of young children. This can relate to intakes of micronutrients such as iron or vitamin D as well as to the balance of macronutrients they consume (e.g. fat or sugar). Alongside this lie concerns regarding overweight and obesity and physical inactivity. This combination of risk factors has potential negative effects for both short and longer term health. Hence, screening tools, such as NutricheQ described here, offer an opportunity for early identification and subsequent appropriate timely intervention from 12 months of age. This paper describes the development and validation of NutricheQ, a short user-friendly questionnaire. Designed to be administered by parents or carers, it aims to help healthcare professionals identify children at risk based on known, evidence-based nutritional risk factors. It is hoped in the longer term that this tool can be adapted for use globally and improve child health through early identification, which can be followed up by targeted, cost-effective interventions.

Dietary surveys from several countries show that the nutritional intake of many very young children fails to comply with dietary recommendations (1–4). Deficits are most commonly reported in relation to nutrients such as iron and vitamin D (5, 6), whereas the early emergence of overweight and obesity, now estimated to affect 40 million preschool children worldwide (7) has been associated with a shift towards energy-rich, nutrient-poor diets (8). This is clearly a public health issue, since early nutrition inadequacies or excesses can exert lasting effects on development (9, 10) and later risk of obesity and related health problems (11, 12). Moreover, dietary habits and preferences formed in childhood may persist into adult life (13). This makes it imperative that parents know how best to manage food fussiness, neophobia and challenging behaviour related to mealtimes that typically present in this phase, since incorrect strategies may exacerbate rather than solve problems (14–16). Despite this, few public health initiatives have been utilised to identify and address feeding and nutritional problems in this formative preschool phase, with both parents and healthcare providers reporting little support and training in this area (17). This suggests a need to identify modifiable risk factors associated with poor dietary quality, inappropriate feeding patterns and imbalanced body weight status during this life stage with screening of nutritional risk recommended (18).

Dietary risk has been defined as any inappropriate dietary pattern that may impair health (19). Short dietary questionnaires or tools offer an attractive means of quickly assessing risk factors for eating patterns that are potentially inadequate, obesogenic, or both. Whereas many of the tools developed in recent years have been designed to identify nutritional risk in sick, hospitalised children or have focused on individual dietary components or food groups (20–24), few have been developed to screen for nutritional risk in healthy preschoolers. One tool, NutriSTEP, designed originally for children aged 3–5 years in Canada, has been adapted for use in younger children (i.e. from 18 months) (25). This tool combines both dietary and behavioural assessments to give one overall score of nutritional risk (25). More recently, a tool which provides a short alternative to a food frequency questionnaire as a means of determining dietary risk in toddlers has been developed (26). However, in light of a clear need for improvement in feeding practices and early nutrition experience, the current study aimed to develop and test a short questionnaire to help healthcare professionals quickly identify known, evidence-based risk factors for dietary deficiencies or excesses in preschoolers aged 12 months plus, to which the parent and healthcare professional can respond. This paper describes the development and subsequent validation of this questionnaire, NutricheQ.

## Methods

**Fig. 1:**
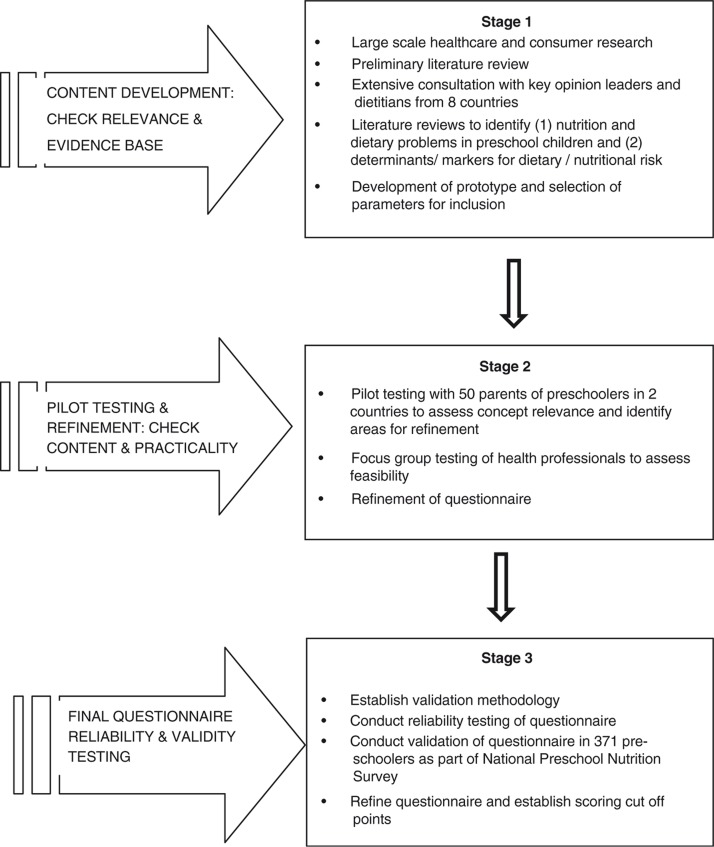
Overview of the development and validation process of NutricheQ.

The development and refinement of the NutricheQ questionnaire is outlined below, followed by a description of the validation study which was conducted in a sample of preschool children who took part in the nationally representative Irish National Preschool Nutrition Survey (NPNS) in 2010 and 2011 (27). An overview of all of the stages involved is illustrated in Fig. 1 and is described below.

### Questionnaire development and refinement

#### Content development and pilot testing

NutricheQ was developed in three stages (Fig. 1). Stage 1 involved healthcare and consumer research, an initial literature review and consultation with international paediatricians and nutrition experts to identify the constructs of nutritional risk for possible inclusion and the criteria for use in a busy clinical setting. This was followed by an extensive literature review, from which constructs were selected for the evaluation of short-term nutritional risk in preschoolers. These were restricted to those with a direct effect on current dietary intake (i.e. types, amounts, and frequency of foods and drinks consumed). Factors relating to the eating environment, developmental aspects of feeding and parental feeding styles were included to attempt to identify potential future risks arising from inappropriate practices, independent of current dietary intake. As there is no reliable means of screening for growth or physical activity levels in very young children other than by direct measurement (28), no questions relating to either were included.

Stage 2 involved a combination of focus groups and pilot testing in two countries (Ireland and Italy) to evaluate feasibility and to assess concept relevance and identify areas for refinement. During the pilot testing, the results of the questionnaire were compared with a full clinical and dietary risk assessment by a paediatric dietitian conducted immediately after completion of the NutricheQ. Following this, further adjustments were made to the questionnaire. Specifically, it became apparent that questions focusing on risk factors for problems that may not emerge until later in life (Section 3 of the questionnaire) could not be directly validated against analysis of nutritional risk based on actual intakes, anthropometric measurements or clinical assessment. While these questions were retained based on robust face validity (29), results from this section were excluded from the below validation testing.

The final NutricheQ administered in the validation study (stage 3) within the NPNS was a three-part, 18-item questionnaire that took between 3 and 5 min to complete. Within the final questionnaire, Section 1 (questions 1–4) aimed to identify risk factors for inadequate iron and vitamin D status, given their relative importance and prevalence in this age group, whereas Section 2 (questions 5–11) focused on risk factors for other dietary imbalances associated with consumption of more energy-dense, nutrient-poor foods and drinks and fewer fruits and vegetables. As mentioned, Section 3 was designed to identify risk factors for longer term nutritional problems arising from poor dietary habit development, inappropriate feeding practices and behaviours (questions 12–18). To facilitate completion and scoring, the number of possible responses per question was limited to three (a, b, c); with answers in the ‘a’ category deemed appropriate or desirable (score of 0), ‘b’ less than ideal (score of 1), and ‘c’ indicating a potential cause for concern/action (score of 2). All items in the questionnaire were scored equally, given the absence of data regarding the relative contribution to dietary risk of different items. The maximum total score obtainable was 22 from 11 questions in Sections 1 and 2. Details of the NutricheQ questionnaire as administered is shown in Table 1. Prior to validation, principal component analysis (PCA) was used to identify underlying components in the questionnaire, whereas Cronbach's alpha was used as a test of reliability (Stage 3) (30). The ‘Cronbach's alpha if deleted’ approach was subsequently applied to identify those questions which were reducing reliability.

**Table 1:** Details of items asked in NutricheQ questionnaire

Section 1
1. My toddler first moved onto cow's milk as his main milk drink (at what age)
2. My toddler usually drinks the following amount of milk, in total, each day (including any used on cereals)
3. My toddler usually eats ‘red’ meat (not including chicken or poultry) OR oily or dark fish
4. My toddler usually eats cereal fortified with iron and vitamins
Section 2
5. I avoid giving my toddler certain foods because of allergy or a food intolerance
6. My toddler eats plenty of fruit (not counting fruit juice)
7. My toddler eats plenty of vegetables
8. My toddler usually has dairy products, including milk (e.g. milk, cheese, yoghurt, fromage frais, milk pudding, custard)
9. My toddler may have more ‘convenience/fast food’ than he/she probably should or than I would like (e.g., chips, burgers, sausages, chicken nuggets, fried rice or noodles, whether home prepared or takeaway)
10. My toddler may have more treats than he/she probably should or than I would like (e.g. chocolate, sweets, biscuits, ice-cream, crisps, other salty snack foods)
11. My toddler usually drinks fruit juice, squash or other sweetened drink (If you add water to juice, only count the juice. Don't include sugar-free drinks)
Section 3
12. My toddler drinks from a bottle (how often per day)
13. My toddler has had difficulty transitioning from smooth textures and has swallowing/chewing problems that concern me
14. I have to be especially careful to control how much my toddler eats, or he would tend to eat too much
15. I use treats and desserts to reward my toddler for good behaviour (or withhold treats if he doesn't behave or finish his meal)
16. My toddler generally has a regular ‘3 meals and mid meal snacks’ routine with meals eaten at table with me/others
17. Mealtimes with my toddler tend to last (the following length of time)
18. My toddler sees me eating healthy meals most or every day with plenty of fruit and vegetables

Each question has three possible responses, a, b, and c with answers in the ‘a’ category having a minimum score of 0 (minimal risk) and answers in the ‘c’ category having a maximum score of 2 (indicating a potential cause for concern). The intermediate category ‘b’ was classed as ‘less than ideal’ with a score of 1. Questions 1–4 relate to Section 1 focusing on iron and vitamin D status, questions 5–11 correspond to Section 2 focusing on risk factors for other dietary imbalances, and questions 12–18 relate to Section 3, which aimed to identify risk factors for longer term nutritional risk. For each section, the question responses are summed with an increased overall score indicating increased risk. As this validation study focused on Sections 1 and 2, the maximum score for these two sections was 22.

### Validation study

#### Study design

NutricheQ was administered to participants of the Irish NPNS (validation study, Stage 3) conducted by University College Dublin (UCD) and University College Cork (UCC) as part of the Irish Universities Nutrition Alliance (www.iuna.net). NutricheQ scores were then compared with relevant food, nutrient, and anthropometric and lifestyle parameters collected in the NPNS by trained researchers as outlined below. Ethical approval was obtained from the Clinical and the Human Ethics Research Committees of UCC and UCD, respectively, and informed consent was obtained in accordance with the Helsinki declaration.

#### Study group

Of the 500 healthy preschool children that participated in the NPNS survey, 371 were aged between 12 and 36 months and included in the validation study. Details of the sampling process, which involved recruitment through a database of young children in the Republic of Ireland (available from EU mom, www.eumom.ie) and childcare facilities, are described elsewhere (27, 31).

#### Data collection

Dietary intake was determined using a 4-day weighed food diary and assessed using WISP^©^ V3.0 (Tinuviel Software, Anglesey, UK), which uses data from McCance and Widdowson's Composition of Foods (6th edition) plus supplements (27). Modifications were made to account for composite dishes, nutritional supplements, generic Irish foods, and new foods on the market (including infant/toddler foods and milks) using Irish food composition data (32). Weight and height (length) of the children were measured by qualified nutritionists, and corresponding *z*-scores calculated for weight, height and BMI as age appropriate (33). Prevalence of overweight and obesity in children aged ≥2 years was calculated using UK WHO age-and-gender specific BMI charts (34) and cut-offs at ≥91st and ≤98th percentile and ≥98th percentile, respectively. The NutricheQ questionnaire was completed by the parents/carer of the child on the researcher's final visit to the participant's home and results from eligible subjects were entered into Q-Builder V2.0^©^ (Tinuviel Software). Quality control procedures (e.g. dual entry) were implemented throughout the collection, processing, and compilation of data.

#### Comparison of NutricheQ scores with NPNS data

The ability of Sections 1 and 2 of NutricheQ (both individually and combined) to evaluate dietary risk was assessed by comparing NutricheQ scores with relevant data collected in the NPNS using three approaches. First, correlation analysis was used to evaluate the relationship between NutricheQ item and section scores with mean daily nutrient intakes. Secondly, quartile analysis was used to determine if higher NutricheQ scores were associated with less nutrient-dense diets and/or higher prevalence of overweight/obesity. This involved dividing the study population into four groups based on NutricheQ total scores and comparing differences in food (g/day), nutrient (µg or mg/10 MJ/per day), and anthropometrics across the quartiles. Thirdly, NutricheQ scores for each child were compared with an objective determination of risk based on dietary intake and anthropometric data using receiver-operating characteristic (ROC) curves. As there is no gold standard for the determination of dietary risk and no suitable food-based index for children younger than 2 years (35), the criteria and cut-off points used were established by an advisory panel of eight expert dietitian and nutritionists taking into account official dietary recommendations and the literature. In brief, risk scores were ascribed to 1) intakes of key nutrients below the Lower Reference Nutrient Intake (LRNI), Estimated Average Requirements (EAR), or Reference Nutrient Intakes; 2) intakes of non-milk sugars, saturated fatty acids, sodium, dietary fibre and total fruit and vegetables in relation to official guidelines, or the range of intakes within the study population; and c) a classification of overweight or obese based on *z*-scores for BMI in children ≥2 years, or weight/length in children aged <2 years weighted accordingly. EAR as established by the Department of Health (UK) (36) were used to determine the proportion of children with inadequate intakes of micronutrients, having been found to be effective in obtaining a realistic estimate of prevalence of dietary inadequacy (37, 38). Where the majority of the study population failed to meet the EAR (e.g. vitamin D), or where no EAR exists (e.g. for intake of fruit and vegetables), cut-off points were established based at the extremes of intakes within the study population, as considered appropriate (see Supplementary Table 1). Using this approach, the total scores for each child ranged from 0 to 34, which were subsequently categorised into one of four risk groups as follows; 1) high risk (score≥16; 9% population), 2) moderate risk (score of ≥8 to ≤15; 35% population), 3) low risk (score of ≥4 to ≤7; 27% population), or 4) negligible risk indicative of desirable intakes relative to guidelines (score ≤3; 29% population). This method was considered more objective than might have been obtained from a rating based on clinical judgement, since it allowed direct comparison of NutricheQ scores with a rating based on detailed nutritional analysis of dietary intake and anthropometric measurement, which could be applied universally in an objective retrospective manner with minimal interobserver bias. ROC curves comparing the two scores (NutricheQ vs. objective criteria) were constructed, being the preferred methodology for establishing validity and informing the selection of the most appropriate cut-off points for risk rating based on sensitivity and specificity at different scores (39). Curves were constructed for the cut-off points for high and moderate number of risk factors and the area under the curve (AUC) measured for both.

#### Under-reporting

Data were analysed including and excluding under-reporters. Minimum energy intake (EI) cut-off points, calculated as multiples of basal metabolic rate, were used to identify under-reporters of energy (40, 41). Data shown include under-reporters (24%) as their removal did not change the overall trends observed.

### Statistical analysis

Descriptive analysis of the study population included mean and standard deviations according to sex, age group, anthropometrics, social class and education. Statistical differences in population descriptives were detected either using one-way analysis of variance or chi-squared tests as appropriate. Pearson moment correlation analysis was used to study the relationship between risk scores for Sections 1 and 2 and their combined scores with mean daily intake of a number of nutrients, fruit and vegetables. Statistical differences in nutrient density, anthropometrics and food group intake were evaluated across quartiles of the NutricheQ score by analysis of covariance (ANCOVA) adjusting for age where necessary and using Bonferroni and Tukey's tests *post hoc* as appropriate. Trend analysis was also evaluated using polynomial contrast. ROC curves and Spearman's correlation coefficient were used to compare NutricheQ scores with objective risk ratings. All statistical analyses were carried out using PASW SPSS^®^ for Windows^TM^ statistical software package version 18.0 (SPSS, Inc., Chicago, IL, USA).

## Results

### NutricheQ reliability

PCA analysis identified that NutricheQ comprised five underlying constructs suggesting it as a multidimensional rather than unidimensional questionnaire. Evaluation of Cronbach's alpha subsequently returned a relatively low score of 0.5; however, it has been reported that values of 0.5 are satisfactory for a multidimensional tool with fewer than 20 questions (42) as is the case with NutricheQ. Furthermore, as dietary quality is known to be a complex and multidimensional construct (35) and as alpha is a function of the number of items in a construct (43), a high Cronbach's alpha value may be unrealistic. The Cronbach's alpha once deleted procedure showed improvements in alpha score following the removal of questions 2 (relating to type and amount of milk consumed) and 5 (relating to the avoidance of one or more food types). However, it was decided to retain these questions on the basis of validation results and a satisfactory evidence base to support their inclusion (i.e. face validity) (29). Furthermore, the subsequent validation analysis was not affected by their inclusion or exclusion.

### NutricheQ validity

#### Study group characteristics

Table 2 describes the study sample which was found to be generally representative of gender and urban/rural location when compared to Census 2006. Of the 248 children aged 2 years or more for whom BMI was calculated, 30% were classified as overweight or obese and none as underweight. Full details of dietary intakes in this cohort are described elsewhere (27). Within the NPNS, most children had adequate micronutrient intakes with the exception of vitamin D and iron and to a lesser extent vitamin A and zinc (44, 45).

**Table 2:** Descriptive characteristics of Irish preschool children aged 1–3 years from the National Preschool Nutrition Survey who participated in the NutricheQ validation study

	Total population	1 year	2 years	3 years	*p*†
Number of participants (n)	371	123	122	126	
Gender (%), male: female	50:50	50:50	52:48	49:51	0.929
	Mean (SD)	Mean (SD)	Mean (SD)	Mean (SD)	
Anthropometrics					
Weight (kg)‡	14.3 (2.8)	11.9 (1.7)	14.2 (1.9)	16.7 (2.2)	0.001
Height (cm)§	91.1 (8.4)	82.6 (4.6)	91 (5.2)	99.2 (4.7)	0.001
BMI (kg/m^2^)‡	17 (2.0)	17.4 (1.8)	17.1 (1.3)	16.9 (1.3)	0.759
Weight z-scores WHO‡	1.6 (1.1)	2.0^a^ (1.2)	1.43^b^ (1.0)	1.28^c^ (1.0)	0.001
Height z-scores WHO§	1.8 (1.8)	3.10^a^ (1.9)	1.45^b^ (1.6)	0.98^c^ (1.2)	0.001
BMI z-scores WHO‡	0.84 (1.1)	0.5^a^ (1.4)	1.05^b^ (0.9)	1.01^c^ (0.9)	0.001
WHO centiles (%)?					
Normal (≤91st centile)	70	−	73	68	0.565
Overweight (>91st to ≤98th centile)	21	−	20	21	
Obese (>98th centile)	9	−	7	11	
Social class (%)¶					
Professional/managerial	64	74	64	57	0.108
Non-manual	15	13	13	19	
Skilled manual	14	7	16	19	
Semi-skilled and unskilled	6	6	7	6	
Education (%)					
Primary/intermediate	5	2	7	6	0.219
Secondary	13	12	9	17	
Tertiary	82	85	84	78	

† One-way analysis/chi-squared tests.

‡ Four participants missing.

§ Five participants missing.

? Aged ≥2 years, *n*=246 (two participants missing).

¶ Three participants missing.

abc Unlike superscript significantly different from each other.

#### Comparison of NutricheQ results with NPNS data

When completed by NPNS participants, the mean NutricheQ scores obtained for Sections 1, 2 and for the total NutricheQ score (i.e. Sections 1 and 2 combined) were 2.7 (SD 1.4), 3.1 (SD 1.9), and 6 (SD 2.4), respectively. Correlation analysis for Section 1 revealed statistically significant, albeit weak (range −0.122 to −0.360, *p*<0.05), negative correlations between NutricheQ scores and seven nutrients (iron, vitamin D, zinc, thiamin, vitamin C, fibre, and saturated fat) and vegetables, the strongest correlation being for iron (−0.36) and vitamin D (−0.331), in which the section was designed to evaluate. For Section 2, statistically significant correlations (range: −0.105 to −0.396, *p*<0.05) were obtained for 14 nutrients (protein, fibre, saturated fat, non-milk sugars, iron, zinc, calcium, riboflavin, folate, thiamin, phosphorous, potassium, carotene, and retinol) and for fruit and vegetables. When scores for both sections were combined (i.e. total score), similar statistically significant weak correlations (range: −0.390 to 0.119, *p*<0.05) were maintained except for saturated fat and vitamin C (Table 3).

**Table 3:** Pearson's correlations between mean daily nutrient intake, mean daily intake of fruit and vegetables, and NutricheQ score for Sections 1 and 2 and for the total NutricheQ scores (Sections 1 and 2)

	Section 1	Section 2	Total NutricheQ score (Sections 1 and 2)
Energy (MJ)	0.016	−0.040	−0.024
Protein (g/day)	0.057	−0.199**	−0.132*
Dietary fibre (g/day)	−0.312**	−0.254**	−0.271**
Total fat (g/day)	0.061	−0.059	−0.015
Saturated fatty acids (g/day)	0.139**	−0.125*	−0.026
Total sugars (g/day)	−0.024	−0.010	−0.020
Non-milk sugars (g/day)	0.057	0.117*	0.119*
Iron (mg/day)	−0.360**	−0.152**	−0.309**
Vitamin D (µg/day)	−0.331**	−0.085	−0.236**
Zinc (mg/day)	−0.234**	−0.233**	−0.308**
Calcium (mg/day)	0.054	−0.300**	−0.214**
Sodium (mg/day)	0.094	0.050	0.085
Riboflavin (mg/day)	0.028	−0.269**	−0.205**
Niacin (mg/day)	−0.202	−0.060	−0.153**
Folate (µg/day)	−0.020	−0.186**	−0.157**
Thiamin (mg/day)	−0.122**	−0.105**	−0.147**
Vitamin C (mg/day)	−0.125*	−0.019	−0.082
Phosphorous (mg/day)	0.105	−0.264**	−0.159**
Potassium (mg/day)	0.071	−0.275**	−0.189**
Carotene (µg/day)	−0.101	−0.251**	−0.283**
Retinol (µg/day)	−0.071	−0.178**	−0.179**
Total fruit (g)	−0.050	−0.199**	−0.191**
Total veg. (g)	−0.129*	−0.396**	−0.390**

* Significant at *p*<0.05 level;

** significant at the *p*<0.01 level, Pearson's correlation coefficient.

Table 4 displays differences in nutrient density across the quartiles of NutricheQ total score. Values ranged from 0–3 in the lowest scoring group to 8–13 in the highest scoring quartile. Analysis of energy-adjusted dietary intakes across the groups showed significant differences in mean daily intakes of most nutrients (*p*<0.05, one-way ANCOVA adjusted for age). In general, nutrient density was significantly lower for those with higher NutricheQ scores, for example, differences between the lowest and highest scoring groups were observed for dietary fibre (27.7±6.7 vs. 21.4±6.8 g/10 MJ/day, *p*=0.001), iron (18.0±6.0 vs. 13.2±4.4 mg/10 MJ/day, *p*=0.001), vitamin D (8.4±7.9 vs. 4.4±5.1 µg/10 MJ/day, *p*=0.002), and carotene (6022.3±6329 vs. 2632±2877 µg/10 MJ/day, *p*=0.001). These patterns were supported by food group analysis where children in the highest scoring groups also ate significantly (*p*<0.05) less vegetables and vegetable dishes, fish/fish dishes and meat, and more non-milk beverages, processed foods and ‘sugars, confectionery, preserves and savoury snacks’ (Table 5).

**Table 4:** Mean daily intakes of nutrients [% total energy (TE), mg or µg/10 MJ/day] for Irish children from the National Preschool Nutrition Survey by quartiles of total NutricheQ score (Sections 1 and 2)

	Quartile 1		Quartile 2		Quartile 3		Quartile 4		p*	Trend
Total NutricheQ score (Sections 1 and 2)	0–3		4–5		6–7		8–13			
*N*	74		104		96		87			
Gender (male:female)	43:57		49:51		50:50		57:43		0.344	

	Mean	(SD)	Mean	(SD)	Mean	(SD)	Mean	(SD)		

Age (years)	1.70^a^	0.79	1.95^a^	0.83	1.96^a^	0.81	2.36^b^	0.73	0.001	
Nutrient analysis										
Energy (MJ)	4.6	1.1	4.6	1.1	4.5	0.9	4.6	1.0	0.723	0.266
Protein (% TE)	16.0^a^	2.8	15.4^ab^	2.4	15.2^ab^	2.0	14.5^b^	2.5	0.009	0.001
Carbohydrate (% TE)	47.8	5.5	49.1	6.1	49.4	5.8	49.1	5.9	0.473	0.583
Dietary fibre (g/10 MJ)	27.7^a^	6.7	25.0^b^	7.2	24.7^b^	6.6	21.4^c^	6.8	0.001	0.001
Total fat (% TE)	34.5	4.6	34.0	5.3	33.8	5.3	34.7	5.2	0.300	0.456
Sat. fat (% TE)	15.9	3.4	15.8	3.6	15.4	3.4	15.6	3.7	0.816	0.885
Total sugars (% TE)	24.8	4.5	25.9	6.0	26.1	5.6	24.8	6.3	0.282	0.515
Non-milk sugars (% TE)	14.0^a^	5.1	16.1^ab^	5.3	17.1^b^	6.3	16.9^ab^	7.0	0.032	0.026
Iron (mg/10 MJ)	18.0^a^	6.0	16.3^ab^	5.6	15.1^bc^	5.6	13.2^c^	4.4	0.001	0.001
Vitamin D (µg/10 MJ)	8.4^a^	7.9	6.4^ab^	6.9	5.2^b^	5.9	4.4^b^	5.1	0.002	0.001
Zinc (mg/10 MJ)	13.1^a^	3.4	11.6^b^	2.6	10.9^bc^	2.7	10.0^c^	2.3	0.001	0.001
Calcium (mg/10 MJ)	1935.1^a^	513.3	1771.1^ab^	503.0	1641.9^b^	512.4	1561.6^ab^	571.8	0.012	0.003
Sodium (mg/10 MJ)	2390.3	680.8	2304.9	644.8	2506.4	623.4	2583.3	644.9	0.092	0.274
Vitamin B6 (mg/10 MJ)	2.9	1.0	2.9	0.9	2.8	0.7	2.6	0.7	0.055	0.012
Vitamin B12 (µg/10 MJ)	9.6	5.1	8.7	3.8	8.5	3.5	7.6	3.5	0.209	0.035
Riboflavin (mg/10 MJ)	3.6^a^	1.0	3.4^a^	1.0	3.2^ab^	0.9	2.9^b^	1.0	0.007	0.001
Niacin (mg/10 MJ)	24.5^a^	6.1	24.3^a^	8.4	23.1^ab^	6.5	21.5^b^	7.2	0.003	0.001
Folate (µg/10 MJ)	411.1^a^	169.3	382.0^ab^	154.7	343.6^b^	118.7	340.8^b^	171.4	0.011	0.002
Thiamin (mg/10 MJ)	2.2^ab^	0.4	2.3^a^	1.1	2.1^b^	0.5	2.0^b^	0.5	0.006	0.010
Vitamin C (mg/10 MJ)	172.8	75.6	159.9	81.9	167.1	87.9	153.3	106.3	0.672	0.366
Phosphorous (mg/10 MJ)	1917.7^a^	359.7	1845.0^a^	335.8	1786.0^ab^	330.2	1696.1^b^	359.3	0.041	0.005
Potassium (mg/10 MJ)	3950.1^a^	612.9	3976.0^a^	656.9	3772.5^ab^	631.3	3476.7^b^	744.0	0.001	0.001
Carotene (µg/10 MJ)	6022.3^a^	6329.4	5179.7^ab^	3711.1	3800.9^bc^	3900.4	2631.9^c^	2877.2	0.001	0.001
Retinol (µg/10 MJ)	1020.8^a^	1686.4	676.7^ab^	353.7	579.8^b^	274.8	560.1^b^	390.6	0.008	0.004

* Age-adjusted one-way analysis of covariance followed by Bonferroni's test; chi-squared test (age only);

abc Unlike superscript significantly different from each other.

**Table 5:** Mean daily food group intake (g/day) for Irish children from the National Preschool Nutrition Survey by quartiles of total NutricheQ score (Sections 1 and 2)

	Quartile 1		Quartile 2		Quartile 3		Quartile 4		
Total NutricheQ score (Sections 1 and 2)	0–3		4–5		6–7		8–13		
*N*	74		104		96		87		

Food groups	Mean	(SD)	Mean	(SD)	Mean	(SD)	Mean	(SD)	p*
		
	(g)/day		(g)/day		(g)/day		(g)/day		

Grains, rice, pasta, and savouries	37.50	31.82	33.86	32.84	34.70	44.30	37.53	36.04	0.844
Bread and rolls	36.92	29.50	39.02	26.58	45.83	31.34	45.05	31.31	0.304
Ready to eat breakfast cereals (RTEBC)	20.40	13.64	23.13	26.68	18.13	14.43	17.32	14.74	0.078
Other breakfast cereals	24.87	35.44	26.94	43.45	17.40	37.73	20.86	51.81	0.441
Biscuits, cakes, and pastries	13.84	12.16	18.55	17.05	17.20	16.55	19.32	15.75	0.222
Infant milk	123.82^a^	199.03	67.86^ab^	157.28	42.74^b^	125.86	22.23^b^	100.95	0.006
Cow's milk	244.94	181.91	270.62	198.88	251.13	208.64	212.55	193.21	0.434
Other milk	11.63	53.75	33.70	117.03	13.69	44.98	28.60	92.82	0.100
Yoghurt and fromage frais	65.09	52.95	55.56	44.61	64.48	58.00	66.52	57.92	0.464
Creams, icecreams, and chilled desserts	15.56	22.15	19.06	26.87	21.42	36.85	15.96	22.14	0.418
Cheese	10.52	12.49	7.23	10.23	7.89	8.36	8.30	9.39	0.166
Butter, spreading fats, and oils	4.75	6.61	4.34	3.60	5.03	4.36	5.59	6.00	0.690
Egg and egg dishes	8.12	15.71	7.69	11.71	8.88	12.34	6.86	11.53	0.643
Potatoes	32.59^a^	41.23	26.29^ab^	26.36	26.88^ab^	27.36	18.98^b^	24.73	0.039
Vegetables and vegetable dishes	48.59^a^	46.93	38.51^ab^	33.13	31.25^bc^	28.33	23.29^c^	21.73	0.001
Fruit	140.88	78.40	138.70	91.20	142.29	90.37	92.87	82.29	0.393
Fish and fish dishes	17.60^a^	22.71	10.10^b^	14.88	9.53^b^	14.63	9.76^ab^	14.61	0.012
Processed foods	18.19^a^	25.25	26.33^a^	30.32	31.05^ab^	28.33	42.49^b^	35.52	0.001
Meat	72.86^a^	63.82	66.55^ab^	51.69	52.09^ab^	36.39	48.05^b^	46.17	0.007
Non-milk beverages†	236.03^a^	167.26	261.25^ab^	181.83	313.94^ab^	218.23	341.05^b^	234.24	0.014
Sugars, confectionery, preserves, and savoury snacks	9.47^a^	11.89	12.08^ab^	13.91	16.74^ab^	16.66	20.20^b^	17.30	0.006
Soups, sauces, and miscellaneous foods	16.38	25.24	19.89	34.41	17.43	30.71	14.04	21.06	0.378
Nutritional supplements	16.98	39.35	13.12	46.14	15.55	39.12	23.24	44.40	0.632
Nuts, seeds, herbs, and spices	0.56	2.08	0.62	2.13	0.34	1.25	0.52	2.31	0.704
Total dairy foods‡	461.42	208.24	441.20	185.91	385.59	208.10	346.43	212.24	0.054

* Age-adjusted one-way analysis of covariance followed by Bonferroni's test;

abc unlike superscript significantly different from each other.

† Includes teas, other beverages, carbonated and diet carbonated beverages, squash, cordials, and fruit juice drinks.

‡ Includes infant milk, cow's milk, other milk, yoghurt and fromage frais, creams, icecreams, and cheese.

#### Comparison of NutricheQ results with objective dietary risk rating

In this cohort, total NutricheQ scores ranged from 0 to 13 from a possible maximum score of 22. When the two risk ratings were compared (i.e. NutricheQ vs. objective criteria), the mean NutricheQ score for preschoolers objectively rated as ‘high’ risk was 8.6 (SD 1.9), ‘moderate’ risk 6.6 (SD 2.3), ‘low–moderate’ risk 5.3 (SD 2.2), and ‘low risk/desirable intake’ 4.1 (SD 1.7), respectively, with correlation analysis returning a moderately strong relationship between the two methods (Spearman's rho=0.53, *p*=0.01).

**Fig. 2:**
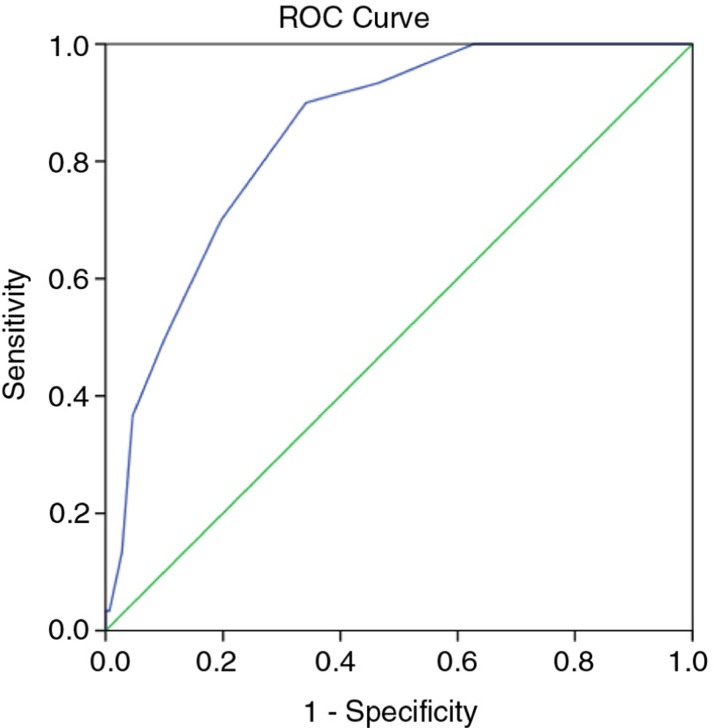
Receiver-operating characteristic (ROC) curve comparing NutricheQ total scores to an objective rating of nutritional risk based on analysis of actual dietary intake and anthropometric measurement using objective criteria. Area under the curve (AUC)=85%.

To further assess the levels of agreement and determine sensitivity (SN) and specificity (SP) across a range of NutricheQ scores, ROC curves were generated based on high and moderate risk ratings using the objective risk criteria. Figure 2 shows the ROC curve for high risk, which has an AUC of 85%, whereas the AUC for moderate risk was 76% (not shown). AUC is generally accepted as the measure of a test or tool's discriminatory power (46), with values of 50% indicating no discriminatory value and 100% indicating a perfect test. AUCs above 75% are generally indicative of clinical value with scores above 80% indicating good discriminatory power. Using the coordinates of the ROC curves to establish the best balance between SN and SP at different NutricheQ scores, cut-off points were proposed at ≥4 (SN 83% and SP 48%) to indicate some areas for improvement and at ≥8 (SN 70%, SP 80%) for multiple areas for improvement. Applying these cut-off points to the NutricheQ scores obtained within the study population placed a majority (56%) into the intermediate risk category, with at least two areas for attention. In contrast, 21 and 23% were placed in the lowest and highest scoring risk categories, respectively. To account for the conservative nature of this approach, the cut-off point for ‘high risk’ was arbitrarily placed at a higher numerical value of ≥10 (*n*=26, 7% of study population, SP 95%) to see if the presence of risk factors continued to increase at higher NutricheQ scores. Comparing groups using cut-offs of ≥8 with those of ≥10, the proportion of children with intakes below the EAR or LRNI increased from 68 to 75% and from 28 to 43%, respectively, and the proportions of overweight and obese children increased from 36 to 43% (data not shown). The proportion of children with intakes of sodium and saturated fats above the 90th percentile was similar (indicating no significant relationship between high sodium/high saturated fat intakes with increasing NutricheQ score) but increased for non-milk sugars (14% for children with a score ≥8 increasing to 22% for children with a score≥10).

## Discussion

This study describes the development and validation of the NutriCheQ questionnaire, a multidimensional tool designed to help healthcare professionals identify dietary risk factors in preschool children. The main study outcome was that this NutricheQ prototype has validity as a means of quickly identifying children at nutritional risk due to less nutrient-dense and/or imbalanced diets. The tool was found to have adequate internal reliability given the brief, multidimensional nature of the questionnaire.

The requirement for a simple dietary risk screening tool, such as NutricheQ, is supported by data showing imbalances in nutrient intakes and nutrient density (1, 6, 7) and an increased prevalence of overweight and obesity in preschoolers (7). In the current study, the majority of Irish children could be broadly classified as having adequate intakes for most nutrients; however, imbalances in dietary quality existed and inadequacies were evident for vitamin D, iron, and dietary fibre and to a lesser extent for vitamin A and zinc. Furthermore, 30% of those aged ≥2 years were either overweight or obese. These figures reflect those reported elsewhere (1, 47, 48). The ability of NutricheQ to identify children at risk in this generally healthy cohort highlights its potential to assist in early identification of modifiable dietary problems, even in populations which could be broadly classified as adequately nourished. Furthermore, as NutricheQ is suitable for assessing risk in children as young as 12 months it can allow for early identification of risk and provision of timely, targeted and cost-effective intervention.

For any tool to be considered for use within a healthcare context, it should ideally be user-friendly, quick to administer, include the major risk factors for a condition and be both reliable and valid (49, 50). The current paper attempted to address these factors. Feedback from health professionals and parents during both pilot and validation testing confirmed the questionnaire to be quick and easy to use, with only 3% of NPNS participants having non-responses for a single question. Given the lack of universally agreed criteria for dietary risk in preschool children, a comprehensive review process during the developmental and pilot stage included contributions from an international expert panel to ensure that the major contributors to dietary risk were addressed by NutricheQ. Cronbach's alpha assessed internal reliability, returning a value of 0.5. Whereas alpha scores of 0.7 are generally recommended (51), the lower alpha score may be explained by the low number of items within a scale (52), tool multi-dimensionality (43), and the inclusion of positive and negative statements as a questioning style. In addition, dietary risk, the central tenet of NutricheQ, comprises many different and often independent factors or items (36), which may not necessarily be expected to correlate as evidenced in other ‘whole diet’ assessments such as Healthy Eating Index 2005 and 2010 where alpha values of 0.59 and 0.61 were returned (35), only marginally higher than that reported here. However, following piloting, the length and style was retained to facilitate easy score computation and speed of administration, both important criteria identified in the early stages of development.

A key element of this validation study involved analysis of relevant nutritional parameters according to NutricheQ score using both quartile and correlation analyses. This approach confirmed that children with higher NutricheQ scores had poorer quality diets. Preschoolers with NutricheQ scores in the highest quartile had less energy-dense diets (*p*<0.05) for a number of macro- and micronutrients, which was generally reflective of higher (*p*>0.05) mean daily intakes of ‘non-core’ foods and drinks including non-milk beverages, ‘sugars, confectionery and savoury snacks’ and processed foods and lower intakes of meat, fish/fish dishes, formula/growing up milk, and vegetables. Nutrient imbalances were also more common in those with high NutricheQ scores; almost half (46%) of those with high NutricheQ scores (>10) had intakes of one or more micronutrients below the UK DH LRNI (excluding vitamin D) versus just 3% in the lowest scoring group (0–3). Furthermore, although this questionnaire did not specifically seek to quantify EI or physical activity, in comparison to children in the lowest scoring group (score of 0–3) where 13% aged ≥2 years were overweight and none obese, almost half (43%) of children with the highest scores (score 10–13, *p*<0.009) were classed as overweight or obese, with higher *z*-scores for weight evident for all age groups (*p*<0.025). Collectively, these results suggest the ability of NutricheQ to identify groups at greater nutritional risk.

The final validation step involved comparison of NutricheQ scores against the objective risk criteria using ROC curves, where the high AUC for the curves (85 and 76%) indicates the validity of the questionnaire and are comparable with that reported elsewhere (25). Two cut-off points of >8 (high risk) and >4 (moderate risk) are suggested for use with associated sensitivities of 70 and 83% and specificities of 80 and 48%, respectively. The sensitivity values were slightly lower than that reported for NutriSTEP (84–92%) (25). This is likely due to three reasons; the use of an objective risk rating based on nutrient intake; the lack of a clinical assessment; and emphasis on specificity in addition to sensitivity due to the quick screening nature of the tool. In this study, a clinical assessment was not feasible hence an objective risk rating was developed. Emphasis was placed on nutrients rather than foods to avoid falsely high levels of agreement while the approach allowed for a detailed and objective means of quantifying nutritional imbalances retrospectively. It also avoided potential errors such as inter-rater variation in risk ratings and bias associated with applying risk to deviations from guidance on portions sizes or servings for good health. This questionnaire should allow healthcare professionals to identify children with NutricheQ scores above the recommended cut-offs and to allow for provision of information and/or referral as required. In instances where children fall below the cut-off scores but clinical concern exists, monitoring should occur, and the child should be referred if concern persists.

Strengths of this study include the rigorous development procedure and the validation against high-quality dietary intake data collected for a large cohort of children as part of a national food consumption survey. Although this study describes the analysis of Sections 1 and 2 combined, when examined independently, Section 1 had a stronger relationship with iron, while Section 2 was stronger for dietary imbalance (data not shown). Hence, each section has the potential for identifying risk in specific areas in an even shorter timeframe. Although Section 3 (which attempted to assess risk factors for the developmental aspects of feeding) could not be validated in this study, this does not indicate that the questions in this section are not useful or should not be included in the tool as a guide or checklist, given satisfactory face validity (29). It does, however, indicate the need for future evaluation of Section 3 and in the interim, as adhered to in this paper, separate assessment and interpretation of scores related to dietary intakes (Sections 1 and 2) to those related to feeding practices and influences (Section 3). Limitations of this study include the rather homogenous population group, and future evaluations should focus on more ethnically diverse populations and children from lower socio-economic groups. Furthermore, given that critical nutrients and their food sources differ between countries, local adaptation to specific questions may be required. Possible adaptations could include 1) substitution of local foods or drinks as examples of the types of items to which a question refers or 2) substitution of locally relevant iron fortified foods for ‘ready-to-use cereals’ (question 4) in countries or regions where foods other than fortified ready-to-use cereals are important contributors to iron intake in preschool children. Further considerations should also assess whether enhanced wording or selective additions to the questionnaire would improve reliability. They should also assess validity in comparison to a clinical assessment and address repeatability as these were not possible in the current study. Finally, this study did not attempt to relate NutricheQ score with biomarkers of nutritional status (due to ethical considerations) or physical activity patterns and growth due to lack of data on physical activity levels in this age group, and the unreliability of parent judgement regarding their young child's growth or weight status (53). In spite of these limitations, NutricheQ was able to identify those children in the NPNS at nutritional risk compared to comprehensive assessments of food and nutrient intake.

## Conclusion

In conclusion, the NutricheQ questionnaire was successful in the identification of preschoolers at nutritional risk in an Irish setting. At a community level, it is hoped that its use will allow healthcare professionals and parents quickly and cheaply identify 1- to 3-year-old preschoolers who are at nutritional risk, thereby facilitating more targeted, cost-effective interventions.

## Supplementary Material

Development and validation testing of a short nutrition questionnaire to identify dietary risk factors in preschoolers aged 12–36 months
