# Supplementary material for: Development and validation testing of a short nutrition questionnaire to identify dietary risk factors in preschoolers aged 12–36 months
Source: Food Nutr Res. 2015 Jun 8;59:10.3402/fnr.v59.27912. doi: 10.3402/fnr.v59.27912 (PMC4461756; doi:10.3402/fnr.v59.27912)
Supplement: Development and validation testing of a short nutrition questionnaire to identify dietary risk factors in preschoolers aged 12–36 months [file FNR-59-27912-s001.pdf]

Supplementary table 1: Criteria for objective risk rating based on dietary and anthropometric data used in NutricheQ validation

Component	Standard for maximum points (High risk)<br><br>Score range 2 – 6	Standard for intermediate points (Medium risk)<br>Score range 1 – 2	Standard for minimum /no points (Low/no risk)<br>Score 0
**Adequacy:**			
Total Fruit (g/d)[a]<br>Total Vegetables (g/d)[b]	<10th percentile*	≥10th to <25th percentile	≥25TH percentile
Dietary fibre (g/10MJ)[c]	<10th percentile	≥10th percentile to <EAR	≥EAR
Vitamins/Minerals (excluding vit. D)[d]	<LRNI	≥LRNI to <EAR / RNI / EFSA RI[e]	≥EAR
Vitamin D (µg/10MJ)[d]	<75% EAR	≥75% EAR to <EAR	≥EAR
**Moderation**			
Sat. Fat (% E)<br>Non Milk Sugars (%E)<br>Sodium (mg/d)	≥90th percentile	n/a	<90th
**Anthropometric data**			
Z score /BMI classification (WHO)[f]	<5th or >98th percentile<br><br>>+or <-3 SD	>91st and ≤98th percentile or ≥5th to <10th<br>≥+2 and <+3 SD	≥10th to ≤ 90th percentile<br><br><+2 and >-2 SD

*Percentiles relate to range of intakes within the study population.
a. Excludes fruit juice. 10th and 25th percentile equivalent to 38g and 56g/day respectively.
b. Includes all forms. 10th and 25th percentile equivalent to 15 and 36g / day respectively.
c. EAR is 2g/10MJ per day. 10th percentile equivalent to 1.3g/10MJ/day.
d. Source: UK Dietary reference values (39).
e. Source: EFSA Panel (2013) ()
f. Source: WHO Obesity and Overweight (2011) ()
EAR, Estimated Average Requirement; LRNI, Lower Reference Nutrient Intake; RNI, Reference Nutrient Intake; EFSA, European Food Safety Authority; E, Energy; BMI, Body Mass Index; WHO, World Health Organisation.
